# Statins, Muscle Disease and Mitochondria

**DOI:** 10.3390/jcm6080075

**Published:** 2017-07-25

**Authors:** Radha Ramachandran, Anthony S. Wierzbicki

**Affiliations:** 1 Departments of Chemical Pathology/Metabolic Medicine, Guys and St Thomas’ Hospitals NHS Foundation Trust, London SE1 7EH, UK; anthony.wierzbicki@kcl.ac.uk; 2 Adult Inherited Metabolic Diseases, Centre for Inherited Metabolic Diseases, Evelina, Guys and St Thomas’ Hospitals NHS Foundation Trust, Lambeth Palace Road, London SE1 7EH, UK

**Keywords:** cardiovascular, statin, myopathy, muscle, mitochondria

## Abstract

Cardiovascular disease (CVD) accounts for >17 million deaths globally every year, and this figure is predicted to rise to >23 million by 2030. Numerous studies have explored the relationship between cholesterol and CVD and there is now consensus that dyslipidaemia is a causal factor in the pathogenesis of atherosclerosis. Statins have become the cornerstone of the management of dyslipidaemia. Statins have proved to have a very good safety profile. The risk of adverse events is small compared to the benefits. Nevertheless, the potential risk of an adverse event occurring must be considered when prescribing and monitoring statin therapy to individual patients. Statin-associated muscle disease (SAMS) is by far the most studied and the most common reason for discontinuation of therapy. The reported incidence varies greatly, ranging between 5% and 29%. Milder disease is common and the more serious form, rhabdomyolysis is far rarer with an incidence of approximately 1 in 10,000. The pathophysiology of, and mechanisms leading to SAMS, are yet to be fully understood. Literature points towards statin-induced mitochondrial dysfunction as the most likely cause of SAMS. However, the exact processes leading to mitochondrial dysfunction are not yet fully understood. This paper details some of the different aetiological hypotheses put forward, focussing particularly on those related to mitochondrial dysfunction.

## 1. Introduction

Cardiovascular disease (CVD) accounts for >17 million deaths globally every year, and this figure is predicted to rise to >23 million by 2030 [1]. Numerous studies have explored the relationship between cholesterol and CVD and there is a consensus that low density lipoprotein cholesterol (LDL-C) is a causal factor in the pathogenesis of atherosclerosis [2,3]. The epidemiological studies underlying this concept have been aggregated and meta-analysed by the Emerging Risk Factors Collaboration [4]. These results provided the impetus for discovery of cholesterol lowering drugs starting with the use of high dose niacin and then proceeding through bile acid sequestrants, fibrates and eventually statins [5,6]. Statins have now become the cornerstone of the management of dyslipidaemia [7].

**Figure 1:**
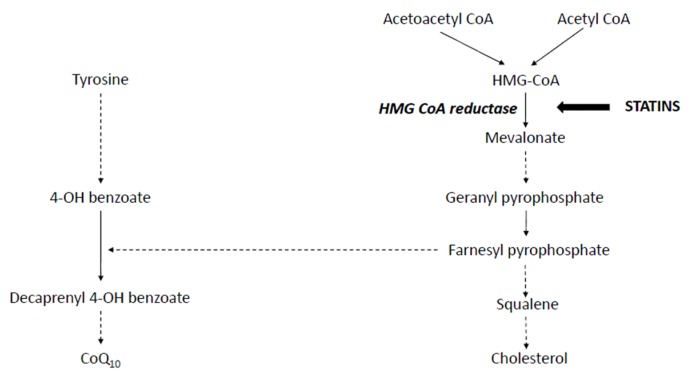
Schematic representation of cholesterol and CoQ10 synthetic pathway. Dotted arrows are used where some of the intermediate products in the pathway have been omitted in the diagram. Site of Statin action is shown. Statin inhibits enzymes HMG CoA reductase which is written in bold italics.

The first step in cholesterol synthesis involves formation of 2-Hydroxymethylglutaryl-coenzyme A (HMG-CoA) by condensation of acetyl CoA and aceto-acetylCoA; HMG-CoA is then converted to Mevalonate by the enzyme HMG-CoA reductase (Figure 1). This is the rate-limiting step in cholesterol synthesis. HMG-CoA reductase was pursued as a viable target for cholesterol lowering drug development. This led to the development of HMG-CoA reductase inhibitors, known as “statins” [6]. Toxicity was limited as HMG-CoA, the immediate precursor before the block, is water soluble and can be metabolised via alternative metabolic pathways, thus preventing accumulation. Numerous attempts have been made to inhibit cholesterol synthesis at other points but these have been limited either by the knowledge of inherited errors of metabolism associated with defects at those sites or by toxicity of potential drug candidates, e.g., squalene synthase inhibition [8].

In September 1987, Lovastatin became the first statin to be given US Food and Drug Administration approval as a cholesterol lowering agent [8]. Two further semi-synthetic (pravastatin and simvastatin) and four synthetic statins (fluvastatin, pitavastatin, atorvastatin and rosuvastatin) of varying efficacy [9] have been successfully introduced into the market since but cerivastatin was withdrawn due to toxicity [10]. The reduction in plasma LDL-C caused by statins is due to upregulation of LDL receptor expression and not only from a decrease in cholesterol synthesis due to HMG-CoA reductase inhibition allied with decreased production of apolipoprotein B containing lipoproteins [11]. These drugs reduce LDL-C levels even in patients with heterozygous Familial Hypercholesterolemia (FH) due to LDL receptor mutations, but not in receptor null homozygous FH [11].

Statins were initially received with some scepticism due to uncertainties regarding benefit and anxieties concerning potential adverse effects [6]. These reservations were dispelled by the results of large long-term randomised controlled trials such as the Scandinavian Simvastatin Survival Study [12]. This study provided unequivocal evidence for reduction in all-cause mortality (30%, *p* = 0.0003), coronary artery related deaths (42%), major coronary events (34%) and revascularisation procedures (37%) with statin therapy. The Heart Protection Study provided further evidence for benefits in women and in patients with diabetes and previous history of cerebrovascular events [13]. Moreover these randomised controlled trials provided reassurance that there was no increase in adverse effects such as cataracts, previously observed in animal studies relating to an earlier cholesterol lowering drug candidate triparanol [14], clinical liver disease [15] or cancer [16], although some concerns continue to be raised [17]. A later patient-based meta-analysis of statin trials showed a 21% reduction in CVD events and an 11% reduction in CV mortality for each 1 mmol/LDL-C reduction [18]. Furthermore, maintaining a 2 mmol/L reduction in LDL cholesterol in 10,000 patients for 5 years prevented approximately 1000 major vascular events in patients with a high risk of coronary events [7,18]. Hence, with good reason, statins are now amongst the most widely prescribed medications across the globe. They are prescribed to roughly 30 million people, and had sales of $25 billion in 2005 [19].

Statins have proved to have a very good safety profile [7,20]. The risk of adverse events is small compared to benefits. Nevertheless, the potential risk of an adverse event occurring must be considered when prescribing and monitoring statin therapy to individual patients. Memory loss, impairment of liver/kidney function, new onset diabetes and muscle symptoms are some of the many adverse effects reported by patients taking statins. Of these, statin-associated muscle disease is by far the most studied [21] and the most common reason for discontinuation of therapy and hence will be the focus of the rest of this paper. 

Presentations with statin intolerance often due to myopathy form up to 10% of the workload of clinical lipid services. Only recently has a classification of statin-related muscle symptoms been agreed [22]. Statin-induced muscle disease can be broadly classified into the rarer, more severe, often irreversible statin-induced necrotising inflammatory myopathy (SINIM) [23], and the relatively more common, reversible spectrum of muscle disease often referred to as statin-associated muscle symptoms (SAMS) [22,24]. There is no consensus about the optimal pathway for investigation of these cases. Investigations include exclusion of common autoimmune muscle diseases and vitamin D deficiency [25]. Vitamin D deficiency can exacerbate statin myopathy but there is no clear evidence for concurrent supplementation having benefits as trials are small [26] and usually not randomised. Some clinicians proceed to muscle biopsy and electron microscopy in severe cases. A case series of 279 biopsies from patients with statin myopathy show a 24% incidence (*n* = 67) of mitochondrial dysfunction on either histochemistry and/or electron microscopy [27]. Ten percent (*n* = 29) had abnormal respiratory chain enzyme activity [27]. 

## 2. Statin-Associated Muscle Symptoms

The reported incidence varies greatly, ranging between 5% and 29% with milder symptoms being common and, the rare, more serious form, rhabdomyolysis being far rarer with an incidence of approximately 1 in 10,000 [17,28,29,30,31]. The Statins on Skeletal Muscle Function and Performance (STOMP) study assessed the effect of 6 months of 80 mg of Atorvastatin on 420 statin-naïve healthy controls and found a significantly increased incidence of muscle-related symptoms in the statin versus placebo group (*p* < 0.05) but found no significant difference in exercise capacity or muscle strength in the statin versus placebo group [32].

Symptoms of SAMS are often non-specific and largely localised to proximal muscle groups such as thighs, buttocks and calves. Physical activity, female gender and Asian ethnicity have all been shown to be associated with an increased risk of SAMS [33,34]. Hypothyroidism, renal and liver impairment and diabetes are other risk factors [35,36]. Interactions due to co-administration of drugs sharing the same cytochrome P450 metabolic pathway may account for up to 60% of SAMS. Drugs such as glucocorticoids, gemfibrozil, protease inhibitors, antipsychotics such as risperidone and immunosuppressives such as cyclosporine, and common food-associated factors such as orange or cranberry juice and excess alcohol consumption have all been implicated [36]. Hence patients must be assessed for these pre-existing risk factors prior to being prescribed statins. 

### 2.1. SAMS Diagnosis

Confirmation of SAMS remains a challenge in the absence of a specific and sensitive biomarker. The 2015 European Atherosclerosis Society Consensus statement suggested that diagnosis should be based on the triad of (i) temporal relationship of symptoms and/or CK elevation to initiation of statin therapy; (ii) disappearance of symptoms on withdrawal; and (iii) re-appearance on re-challenge with statin therapy [36]. Similar proposals were mooted by other expert groups including the Canadian Consensus Working Group [29]. 

### 2.2. SAMS Classification

SAMS can be further classified based on muscle symptoms, the presence and degree of CK elevation [22]. Muscle symptoms with no elevation in CK, often referred to as myalgia, is regarded as the mildest form. The term myositis is sometimes used to describe symptoms associated with significant CK elevation (>10 times upper limit of normal range). Rhabdomyolysis is the most severe form, and may result in myoglobinuria and renal impairment. CK levels in rhabdomyolysis may rise to >40 times upper limit of normal range. 

The pathophysiology of and mechanisms leading to SAMS is yet to be fully understood. The rest of this paper will detail some of the different aetiological hypotheses put forward and, in particular, focus on the hypotheses related to mitochondrial dysfunction. 

### 2.3. SAMS Pathobiology

#### 2.3.1. Genetic Predisposition

Polymorphisms in a number of genes, including those coding for efflux ABC transporters (ABCB1 and ABCG2), influx transporter- organic anion–transporting polypeptide 1B1 (OATP1B1) (*SLCO1B1*) and Cytochrome P450 enzymes: CYP2D6, CYP3A4, and CYP3A5, have been associated with SAMS. However, thus far, only SNPs in the *SLCO1B1*, causing disruption in the hepatic uptake of simvastatin, have shown convincing associations [37]. Routine testing for this polymorphism is currently not recommended. In contrast, polymorphisms leading to reduced expression of *GATM* gene may offer a degree of protection against SAMS [38]. Whilst the exact mechanism is not known, it was proposed that reduced creatine synthesis, and hence reduced phosphocreatine stores, modify cellular energy stores and the AMPK signalling favourably. However, other studies have failed to replicate the results [39]. Furthermore, creatine deficiency related to a loss of function mutation in *GATM* can present with myopathy [38]. Statins precipitate myopathic symptoms in patients with genetic mutations for rare inherited metabolic disorders such as MELAS (Mitochondrial encephalomyopathy, lactic acidosis, and stroke-like episodes) syndrome [40,41,42,43,44]. A study of 110 patients with statin-induced myopathy reported a 4-fold (*p* < 0.001) increase in pathogenic mutant alleles for carnitine palmitoyltransferase II deficiency, McArdle disease and myoadenylate deaminase deficiency versus controls [43]. Despite these isolated reports in the literature, convincing evidence to recommend routine testing for genetic predispositions prior to initiation of treatment with statins remains elusive.

#### 2.3.2. Mitochondrial Dysfunction

Mitochondrial dysfunction is defined as a decrease in the ability of the mitochondria to synthesise high energy compounds such as adenosine 5′ triphosphate and a suboptimal electron transfer rate across the respiratory chain complex. Primary mitochondrial disease results in mitochondrial dysfunction due to mutations in genes coding for mitochondrial function. In addition, mitochondrial dysfunction related to oxidative damage is a recognised feature of aging and a number of chronic diseases including diabetes [44]. 

Mitochondrial dysfunction can also be precipitated by drugs and is now the most widely accepted and studied pathobiological mechanism for SAMS [45,46]. However, the exact nature/extent/type of mitochondrial dysfunction(s) causing SAMS remains unclear. Studies seeking to establish the aetiology of SAMS have demonstrated impairment of numerous mitochondrial processes. Results and hypotheses of some of these studies have been summarised below. 

##### Coenzyme Q_10_ Deficiency

Statin-induced Coenzyme Q_10_ (CoQ_10_)/Ubiquinone deficiency is the most commonly mooted and extensively studied aetiological cause for SAMS. Ubiquinone comprises a quinone ring and a 10 isoprenoid unit side chain. Whilst some of it is derived from diet, a significant proportion, approximately half, is synthesised within the mitochondria (Figure 1) [47,48]. It is present within the inner mitochondrial membrane of eukaryotic cells and is involved in many different metabolic processes including electron transfer in the mitochondrial respiratory chain [49,50]. It is an essential cofactor for a number of dehydrogenases including those involved in fatty acid oxidation and pyrimidine synthesis. It is also an antioxidant and has a role in apoptosis. 

Statins reduce synthesis of a number of other intermediary isoprenoid compounds downstream in the cholesterol biosynthesis pathway [51,52]. Low levels of CoQ_10_ secondary to inhibition of mevalonate synthesis by statins has been implicated in SAMS aetiology. However, many studies have produced conflicting/equivocal conclusions [50]. 

A number of intervention trials with CoQ_10_ have been performed in statin-associated myalgia following anecdotal case reports of benefit. A systematic review and meta-analysis assessed data from 6 randomised control trials with 8 placebo-controlled treatment arms which met the inclusion criteria: (i) placebo controlled study; and (ii) presented sufficient information about change in CoQ 10 levels following statin therapy [50]. This meta-analysis included a total of 240 participants and 210 controls, with duration of trial interval varying between 6 and 26 weeks. The trials included statin therapy with simvastatin (20–80 mg/day), atorvastatin (10–40 mg /day), rosuvastatin 40 mg/day and pravastatin 20 mg/day. The results showed a significant reduction in circulating CoQ_10_ levels (−0.44 umol/L; *p* < 0.001) in the statin versus placebo arms. The degree of reduction was independent of type or dose statin used and duration of study (>12 weeks versus <12 weeks). However, the clinical relevance of the reduced serum/plasma CoQ_10_ levels remains unclear. Most CoQ_10_ is found in LDL particles, so any decrease in measured circulating levels following statin therapy may have been related to lowering in LDL-C levels with little associated change in tissue levels [53]. Indeed, no correlation was found between plasma and muscle CoQ_10_ levels in the 48 patients studied in a randomised controlled trial assessing the effects of statins on cholesterol and CoQ_10_ metabolism [54]. Paiva et al. studied CoQ_10_ levels in skeletal muscle and demonstrated that CoQ_10_ levels were significantly lower in simvastatin-treated patients versus controls but confusingly no difference was observed in patients treated with Atorvastatin [54]. They also measured respiratory chain activity in six of the subjects and observed a concomitant decrease in respiratory chain activity, although the ratio between citrate synthase and complex activities remained unchanged [54]. They therefore hypothesised that lower muscle CoQ_10_ levels seen in simvastatin-treated patients were associated with mitochondrial volume loss, rather than a true decrease in CoQ_10_ levels within the mitochondria [54,55]. 

The reported lack of correlation between plasma and muscle CoQ_10_ levels show that, ideally, CoQ_10_ levels should be measured in the correct sub-compartment [56]. However, measuring CoQ_10_ levels in skeletal muscle involves a muscle biopsy, which is a technically challenging, requires admission, and is significantly more invasive than taking a simple blood sample. CoQ_10_ levels in peripheral blood mononuclear cells have been shown to correlate well with muscle CoQ_10_ levels and may be used as a good, less invasive surrogate marker for tissue CoQ_10_ levels in future studies [47,57]. Using this technique, Avis et al. were able to demonstrate a significant drop in CoQ_10_ levels in muscle and mononuclear cells of children with familial hypocholesterolaemia treated with Rosuvastatin [57]. But they were unable to demonstrate any associated drop in mitochondrial ATP synthesis and hypothesised that a decrease in mitochondrial ATP synthesis may only become apparent when CoQ_10_ levels fall below a certain minimal threshold [57]. The same group have previously reported reduced CoQ_10_ and complex IV levels in muscle biopsy samples from two patients presenting with simvastatin associated rhabdomyolysis [58]. It is of note that both of these patients had other predisposing factors that increase risk of myopathy. They were on medications (cyclosporine and itraconazole) that have been reported to result in increased circulating simvastatin levels on co-administration [59,60]. Furthermore, since the patients presented following rhabdomyolysis, an underlying mitochondrial muscle pathology predating statin therapy could not be confidently excluded [58]. 

There is little evidence to support routine CoQ_10_ measurement and supplementation for statin-related myalgia [29,36,61]. However, assessment of CoQ_10_ function prior to and after starting statin therapy using mononuclear cell levels may be suitable in a small group of patients with suspected inherited deficiencies of CoQ_10_ biosynthesis. The preferred treatment option in susceptible patients with these conditions would be to use alternative lipid lowering therapies such as PCSK9 inhibitors that have recently been approved for prescription in the UK. 

##### Mitochondrial Depletion

A retrospective analysis of the muscle biopsy samples of 48 patients showed a significant decrease in mitochondrial DNA copy number versus nuclear DNA copy numbers (median −47%), thus suggesting mitochondrial depletion in the simvastatin-treated group [54], as did a study of muscle biopsies in 23 patients [62]. The degree of depletion varied, and was independent of clinical signs or symptoms. It was suggested that symptoms may only become apparent once a critical threshold of depletion is reached, similar to other inherited disorders of mitochondrial depletion [63]. A number of different mechanisms for mitochondrial depletion have been proposed. Several studies using rodent myocytes, and human and rodent cell cultures suggest a role for pathways including insulin-like growth factor 1(IGF-1)/Akt in mitochondrial damage and hence apoptosis [64]. Statins have been shown to induce apoptosis by increasing Atrogin-1 mRNA expression in rodent cardiomyocytes [65]. Other studies incriminate decreased mitochondrial biogenesis secondary to statin-induced downregulation of the transcriptional co-activator peroxisome proliferator activating receptor gamma co-activator 1 (PGC1α) as a cause of SAMS [66]. PGC1α upregulates mitochondrial biogenesis by activating nuclear respiratory factor 1, which in turn regulates transcription of transcription factor A. Some studies have reported a reduced PGC1α mRNA expression in human muscle cells on exposure to statins [66,67]. PGC1α also decreases atrophy gene expression, and thus muscle atrophy [68]. A reduced statin-related expression of PGC1α has therefore been proposed as a putative cause of increased atrophy gene expression and muscle atrophy. However, in one study, while muscle atrophy genes were upregulated in statin-exposed muscle, the upregulation was independent of changes in PGC1α or mitochondrial content [69]. Muscle tissue varies in its response to statin exposure. Rodent studies show differential effects on fast versus slow twitch muscle fibres [69,70,71]. Fast twitch fibres appear to be more susceptible to statin related damage. West Africans, who have more fast twitch muscle fibres [72], tend to have higher CK levels and show an increased rate of statin myopathy [36]. In contrast to skeletal muscles, statins appear to protect mitochondria in cardiac myocytes from oxidative stress [73] possibly by activating PGC1α via reactive oxygen species and inducing mitochondrial biogenesis. This has been proposed as a possible hypothesis for the protective effect of statins on the cardiac mitochondria. Studies on cardiac and skeletal muscles of patients on statins have demonstrated that statins generate low levels of ROS in the cardiac muscle of patients, thus promoting mitochondrial biogenesis [50]. This effect is complex, as large concentrations of ROS in the skeletal muscle have the opposite effect on mitochondrial biogenesis [66]. 

##### Inhibition of Mitochondrial Respiratory Chain Complexes

Direct inhibition of one or more complexes in the mitochondrial respiratory chain has been proposed as a possible cause of statin myopathy [27,51]. 

Adverse effects of statins on L6 rat myocyte cell lines and in rodent muscle biopsy specimens include impaired function of complexes I, III and IV of the respiratory chain [74] when exposed to >100 umol/L of cerivastatin, simvastatin, fluvastatin, atorvastatin. No significant toxicity was seen at concentrations of 1 umol/L. Secondary effects of statins included disturbances in mitochondrial membrane potential, fatty acid beta-oxidation, mitochondrial membrane permeability, DNA fragmentation and apoptosis. The effects were more pronounced in the lipophilic statins but were only seen at high concentrations with a hydrophilic statin. Importantly, pravastatin did not impair electron chain activity even at concentrations of 1 mmol/L [74]. This might suggest a relatively lower SAMS rate in pravastatin-treated patients, but this is not been validated in large studies [20].

Sirvent et al. conducted some elegant experiments to assess the effect of simvastatin on human myocytes. They were able to demonstrate respiratory inhibition of complexes I to IV, with the main effect being inhibition of complex I activity. ATP synthase activity (complex V) was not affected [75]. Both lipidic and glucidic pathways were equally effected, showing up to 20% reduction when treated with 50 uM Simvastatin [75].

Whilst these in vitro studies are convincing, it is important to note that the doses of simvastatin used were more than a thousand fold higher than levels achieved in patients treated with therapeutic doses of Simvastatin, where serum concentrations are typically 1–15 nmol/L, and concentrations in muscle are 30% of circulating concentrations [76]. A direct extrapolation of these effects to patient populations is therefore difficult to make. This could account for discordance between cell studies and relatively lower incidence of human adverse event reported by larger meta-analytical studies [20]. Nevertheless, case reports and case cohort studies have reported decreased respiratory chain activity, especially complex IV activity in patients on statin therapy. 

Phillips et al. examined muscle biopsy specimens from four patients who gave history of muscle symptoms on statin therapy. Their symptoms resolved on discontinuation of statin. Biopsy specimens showed red ragged fibres, decreased staining for cytochrome oxidase, and increased lipid droplets. Repeat biopsy done on three patients 3–6 months after discontinuation of therapy showed complete resolution of symptoms. No other comorbidities were found in these patients and their creatine kinase levels remained within reference limits throughout [77]. Similar reports were published by Duncan et al., who showed decreased complex IV activity in addition to decreased CoQ_10_ levels in two patients who presented with rhabdomyolysis when simvastatin was co-administered with another medication (Cyclosporine and Itraconazole) [58]. Arenas et al. reported 60% reduction in cytochrome oxidase activity patient who presented with myoglobinuria on co-administration of cerivastatin and gemfibrozil [78]. They proposed that this was due to cerivastatin related depletion of isoprenoid farnesyl pyrophosphate (FPP) [78,79]. FPP serve as lipid moieties for a number of intracellular compounds and hence can affect a number of intracellular signal pathways and functions including complex IV activity [78,79]. In fact, the role of FPP depletion in a number of proposed beneficial and harmful effects of statins has been extensively studied [79]. It is important to note, however, that cerivastatin has been withdrawn from the market due to significant adverse effects [80]. Safety profile of currently available statins are significantly better that cerivastatin, and hence cerivastatin-mediated effects, cannot automatically be extrapolated to other statins. Furthermore, gemfibrozil is known to increase risk of SAMS when co-administered with statins [36]. 

##### Lactone Toxicity

Statins are converted to lactones from statin acids. Lactones are produced by uridine 5′-diphospho-glucuronosyltransferases, and may be responsible for the cytotoxic effects of statins [81]. Recently a comprehensive study investigated the effect of seven different statins on C2C12 myoblast cell lines [82]. Lactones were more powerful cytotoxic agents than their acid counterparts, and induced cytotoxity through apoptosis. Lactones also acutely decreased mitochondrial ATP generation through inhibition of complex III in the mitochondrial respiratory chain by binding with one of two binding sites involved in electron transfer from CoQ_10_ to cytochrome C. These findings were corroborated in muscle biopsy samples from 37 patients with history of statin-related myopathy. The decrease in complex III activity correlated with both symptoms and with muscle levels of statins. Higher levels of toxicity were observed for more lipophilic statins atorvastatin and simvastatin compared with the hydrophilic statins pravastatin and rosuvastatin [82]. 

##### Impaired Ca^2+^ Homeostasis

Rodent cell culture, rodent in vivo and human myocyte studies have shown impaired excitation-contraction coupling of skeletal muscle fibres in response increased cytosolic Ca^2+^ efflux secondary to altered mitochondrial function as a mechanism for statin-related myopathy [51,83,84]. Guis et al. conducted contraction tests and ^31^P magnetic resonance spectroscopy studies of muscle biopsy specimens taken from nine patients with statin-related myopathy, which showed abnormal contraction in 7/9 patients and delayed proton efflux, suggestive of impaired calcium homeostasis [85]. A pharmacogenetic study of cerivastatin, a highly myopathic statin, noted an association of both increased and decreased adverse events with different ryanodine receptor polymorphisms (RYR2) in 185 patients with rhabdomyolysis [86]. Changes in calcium-release mechanism gene expression occur in patients without myalgia exposed to statins [87]. A 1.56 (95% confidence interval 1.20–2.10) fold increased risk of muscle symptoms or raised CK has been described in 332 patients with malignant hyperthermia and 3261 of their relatives from Sweden. This increases to 52 (22–123)-fold for drug-induced myopathy and a 30 (6–148) fold increase for hyperthermia when compared with 3320 controls and 30,728 relations [88]. Statins may therefore unmask disorders of calcium homeostasis such as malignant hyperthermia. 

##### Substrate Overload

Impaired glucose oxidation secondary to statin-related induction of atrogin-1 mRNA expression causes glycogen accumulation in muscle due to inhibition of pyruvate dehydrogenase complex activity [89]. Statin-induced impairment of beta-oxidation leading to lipid accumulation in the muscle cells has also been reported [69]. Both these mechanisms can potentially lead to eventual development of insulin resistance and then muscle atrophy [90]. 

## 3. Statin-Induced Necrotising Inflammatory Myopathy

This is a rare autoimmune disease related to the presence of anti-HMG CoA reductase antibodies associated with a restricted HLA type (DRB1*11:01) [23,91,92]. Patients with previous statin exposure develop symmetrical proximal myopathy with grossly elevated creatine kinase (CK). Symptoms persist despite cessation of statin therapy. It is very rare, and has a reported incidence of less than 2 per million per year. Muscle biopsy reveals muscle fibre necrosis with minimal endomysial inflammatory infiltrates. Diagnosis is confirmed by the presence of anti-HMG CoA antibodies and characteristic findings on muscle MRI [23]. The mainstay of treatment is cessation of statin therapy and immunosuppression [92]. A detailed review of SINIM is beyond the scope of this paper [23]. 

## 4. Conclusions

Statins are one of the most widely prescribed therapeutic agents because of their proven track record in significantly reducing cardiovascular mortality. Whilst mostly well tolerated, SAMS is the most common cause of statin intolerance and discontinuation of therapy. A number of factors including genetic predispositions and drug interactions have been associated with an increased risk of SAMS. Whilst evidence in the literature points towards statin-induced mitochondrial dysfunction as the most likely cause of SAMS, the exact processes leading to mitochondrial dysfunction are not yet fully understood. Larger and more robust studies looking the plausible pathways are needed to enable a more thorough elucidation of SAMS pathology and to identify biomarkers of risk. 

## Abbreviations

CVD	Cardiovascular disease
SAMS	Statin associated muscle disease
HMG-CoA	2-Hydroxymethylglutaryl-coenzyme A
LDL	Low density lipoprotein
FH	Familial Hypercholesterolemia
CV	Cardiovascular
CK	Creatine Kinase
SINIM	Statin induced necrotising inflammatory myopathy
CoQ10	Coenzyme Q_10_
ATP	Adenosine triphosphate
PCSK9	proprotein convertase subtilisin–kexin type 9
IGF-1	insulin like growth factor 1
PGC1α	peroxisome proliferator activating receptor gamma co-activator 1
ROS	reactive oxygen species
FPP	farnesyl pyrophosphate
RYR2	ryanodine receptor polymorphisms
